# Invasive hematophagous arthropods and associated diseases in a changing world

**DOI:** 10.1186/s13071-023-05887-x

**Published:** 2023-08-17

**Authors:** Ross N. Cuthbert, Frédéric Darriet, Olivier Chabrerie, Jonathan Lenoir, Franck Courchamp, Cecilia Claeys, Vincent Robert, Frédéric Jourdain, Romain Ulmer, Christophe Diagne, Diego Ayala, Frédéric Simard, Serge Morand, David Renault

**Affiliations:** 1 Institute for Global Food Security, School of Biological Sciences, Queen’s University Belfast, Belfast, UK; 2 MIVEGEC, Université Montpellier, IRD, CNRS, Montpellier, France; 3 UMR CNRS 7058 “Ecologie et Dynamique des Systèmes Anthropisés” (EDYSAN), Université de Picardie Jules Verne, 1 rue des Louvels, 80037 Amiens Cedex 1, France; 4 Ecologie Systématique Evolution, Université Paris-Saclay, CNRS, AgroParisTech, Gif sur Yvette, France; 5 Centre de Recherche sur les Sociétés et les Environnement Méditerranéens (CRESEM), UR 7397 UPVD, Université de Perpignan, Perpignan, France; 6 Santé Publique France, Saint-Maurice, France; 7 CBGP, Université Montpellier, CIRAD, INRAE, Institut Agro, IRD, 755 Avenue du Campus Agropolis, 34988 Cedex, Montferrier-Sur-Lez France; 8 Medical Entomology Unit, Institut Pasteur de Madagascar, BP 1274 Antananarivo, Madagascar; 9 Faculty of Veterinary Technology, CNRS - CIRAD, Kasetsart University, Bangkok, Thailand; 10 Université de Rennes, CNRS, ECOBIO (Ecosystèmes, Biodiversité, Évolution) - UMR 6553, Rennes, France; 11 Institut Universitaire de France, 1 Rue Descartes, Paris, France

**Keywords:** Anthropogenic activities, Biological invasion, Biodiversity homogenization, Climate change, Global trade, Public health, Mosquitoes, Ticks

## Abstract

Biological invasions have increased significantly with the tremendous growth of international trade and transport. Hematophagous arthropods can be vectors of infectious and potentially lethal pathogens and parasites, thus constituting a growing threat to humans—especially when associated with biological invasions. Today, several major vector-borne diseases, currently described as emerging or re-emerging, are expanding in a world dominated by climate change, land-use change and intensive transportation of humans and goods. In this review, we retrace the historical trajectory of these invasions to better understand their ecological, physiological and genetic drivers and their impacts on ecosystems and human health. We also discuss arthropod management strategies to mitigate future risks by harnessing ecology, public health, economics and social-ethnological considerations. Trade and transport of goods and materials, including vertebrate introductions and worn tires, have historically been important introduction pathways for the most prominent invasive hematophagous arthropods, but sources and pathways are likely to diversify with future globalization. Burgeoning urbanization, climate change and the urban heat island effect are likely to interact to favor invasive hematophagous arthropods and the diseases they can vector. To mitigate future invasions of hematophagous arthropods and novel disease outbreaks, stronger preventative monitoring and transboundary surveillance measures are urgently required. Proactive approaches, such as the use of monitoring and increased engagement in citizen science, would reduce epidemiological and ecological risks and could save millions of lives and billions of dollars spent on arthropod control and disease management. Last, our capacities to manage invasive hematophagous arthropods in a sustainable way for worldwide ecosystems can be improved by promoting interactions among experts of the health sector, stakeholders in environmental issues and policymakers (e.g. the One Health approach) while considering wider social perceptions.

**Graphical abstract:**

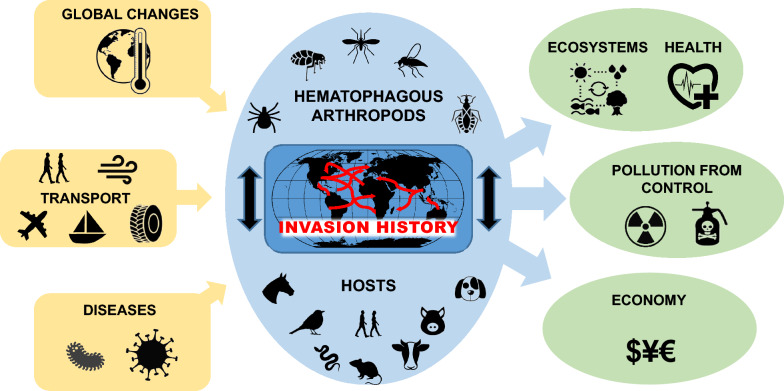

**Supplementary Information:**

The online version contains supplementary material available at 10.1186/s13071-023-05887-x.

## Background

Invasive hematophagous arthropods (those that establish and spread outside of their native range) can be major vectors of pathogens and parasites to animal and human populations. When they spread outside their historical range because of human activities, these blood-feeding arthropods, such as mosquitoes and ticks, can have ecological, economic and social impacts. The pathogens they bear include arthropod-borne viruses—also called arboviruses, belonging to the Flaviviridae, Togaviridae, Reoviridae and Bunyaviridae families—which are responsible for numerous widely distributed illnesses, such as dengue, yellow fever, chikungunya, Zika and West Nile viruses [1]. As such, they give rise to major ecological, economic and health problems worldwide [2–4] and require significant management and surveillance efforts (also see Box 1). In parallel, the world’s economic growth and intensification of international trade and travel exacerbate these threats by promoting the emergence of vector-borne diseases [5, 6]. As is the case for most invasive arthropods and plant species, ongoing climate and land-use changes, as well as other human pressures on the environment, may create new suitable niches for the proliferation of hematophagous arthropods, boost their performances, and thus facilitate their range shifts and establishment beyond their former geographical limits [7–12].

Socioeconomic factors are major drivers facilitating the transport and spread of alien species. For instance, using data collated by DAISIE (Delivering Alien Invasive Species Inventories for Europe, http://www.europe-aliens.org/), Pyšek et al. [13] showed that large industrialized countries in western Europe have harbored the highest numbers of alien populations. That is partly because these countries are historically more interconnected through colonialism and trade and therefore have accrued a larger number of alien invasive species. Moreover, a positive relationship has been observed between national gross domestic product (GDP) and the total number of alien species per country, indicating that economic growth could link to invasion rates [14]. In turn, relationships between economic measures—such as GDP, international trade or research effort—and the economic impact of biological invasions have been reported [15–17]. Often, invasive alien species have tremendous costs to national economies [18–21]; this is particularly true for invasive hematophagous arthropods, which are sources of extremely high healthcare costs [4]. Recent assessments estimated that the economic cost of invasive alien species reached at least US$ 2.168 trillion over the last 4 decades (see Living Figure in [22], and https://borisleroy.com/invacost/invacost_livingfigure.html), with *Aedes* mosquitoes being the most expensive genus among aquatic and semi-aquatic invaders, with a total cost of potentially US$ 311 billion [23]. This economic burden is expected to increase further if the worldwide proliferation and invasion by alien hematophagous arthropods that can vector disease are not halted.

Ongoing climate change may assist the invasion rates of alien species in addition to facilitating range-shifting species expanding beyond their native range (the so-called ‘neo-native’ species [24]; see Box 2 for additional information). It is important to bear in mind that changing climate and increasing travel and trade are interactive forces driving establishment success during introduction and making the invasiveness of alien hematophagous arthropods after introduction even more likely. Hence, the outcomes of complex interactions between climate change and biological invasions require far more consideration [25, 26]. The positive role of climate warming in the establishment rate of alien populations of insects in the period 1900–2005 was demonstrated by Huang et al. [27]. Using species distribution models, Bellard et al. [7] found that, on average, insects belonging to the IUCN’s list “100 of the world’s worst invasive alien species” would increase their suitable range by 18% in 2050. Similarly, invasive alien hematophagous arthropods will likely do so in the future [28, 29].

Owing to the long-standing invasion history of hematophagous arthropods, affecting human populations since the Paleolithic Era [30], their ecological consequences have been well documented [31, 32], and the magnitude of their impact can highly vary according to the species considered. Numerous studies have reported the negative outcomes incurred by these arthropods, including their major impacts on the survival of resident species [33–35], on local human communities [31], as well as on ecosystem functioning and services [36, 37]. Surprisingly, and despite the known health threat of invasive hematophagous arthropods, and their high diversity, our understanding of how global change will impact the population dynamics of existing and emerging invaders—and the epidemiology of the diseases they might transmit—remains poor [38, 39]. Also, there is an urgent need to compile information on the historical and social aspects of hematophagous arthropod invasions to inform policymakers and prepare robust risk mitigation strategies based on lessons learned and sound risk assessment. In this context, this review examines the different actions and roles that invasive hematophagous arthropods, vectors of disease, play in a rapidly changing world, considering a range of socioeconomic and ecological contexts and case studies. By “vector,” we refer to hematophagous arthropods which can spread pathogens and/or parasites that cause disease, belonging to insects (mosquitoes, phlebotomine sand-flies, culicoides, body lice, fleas, etc.) or ticks (hard Ixodidae ticks and soft Argasidae ticks) and mainly consuming blood from terrestrial mammals, birds, reptiles and/or amphibians. Importantly, this review focuses in scope on all invasive hematophagous arthropods, despite differences in their invasion ecology and behavior. In part, this allows us to highlight knowledge gaps in the study of these taxa as well as highlight the diversity of processes through which they are introduced, succeed and cause impact. However, given the uneven research effort that is predominantly targeted towards a few species of mosquitoes and ticks, as well as the expertise of the current authors, these taxa are most prominently exemplified in this review.

Previous reviews have examined the biology, ecology and health impacts for vertebrates as well as risks and management of important groups of invasive hematophagous arthropods and associated diseases [e.g. 38, 38, 38]. Here, our review provides a broader, overarching perspective that spans invasion dynamics, ecological and socioeconomic impacts, global change drivers and wider social and cultural dimensions in an integrative way across multiple taxa. Particularly, this review provides a novel synthesis of the historical contexts underpinning hematophagous arthropod invasions, by amalgamating, for the first time, a chronology of arrivals and pathways for these species and thereby allowing future management strategies to be informed by historical trajectories of past invasion dynamics alongside anticipated socioeconomic and environmental changes. First, we consider past and present invasions of hematophagous arthropods, assuming that ongoing globalization and climate change will drive and accelerate further proliferations in the future. In doing so, we construct a timeline of past disease vector invasions across hematophagous arthropod groups, considering individual species and socioeconomic drivers. Second, we explore the ecological, economic and health impacts of these taxa, anticipating a higher competitive ability of invasive alien species over natives, marked risks to native species through disease vectoring and substantial economic costs to activity sectors such as healthcare. Third, we discuss the combined threat of future climate change and invasions from hematophagous arthropods to human populations. Last, we explore the social-ethnological dimension of these invasions (i.e. we considered how the different cultural contexts and people living in different social contexts could drive the perception of invasive hematophagous arthropods), with the aim to highlight opportunities for collaboration among the health sector and environment researchers, alongside engagement with policymakers and citizens. Ultimately, this will contribute to placing hematophagous arthropod invasions and their management in a broader sustainability context, whereby management strategies considered by scientists, public health officials and communities are considered in tandem with, and do not compromise, environment quality, ecosystem services and conservation values.

## Chrono**-**geography of invasions

### Mosquito invasions in the Renaissance period and during European expeditions

**Fig. 1:**
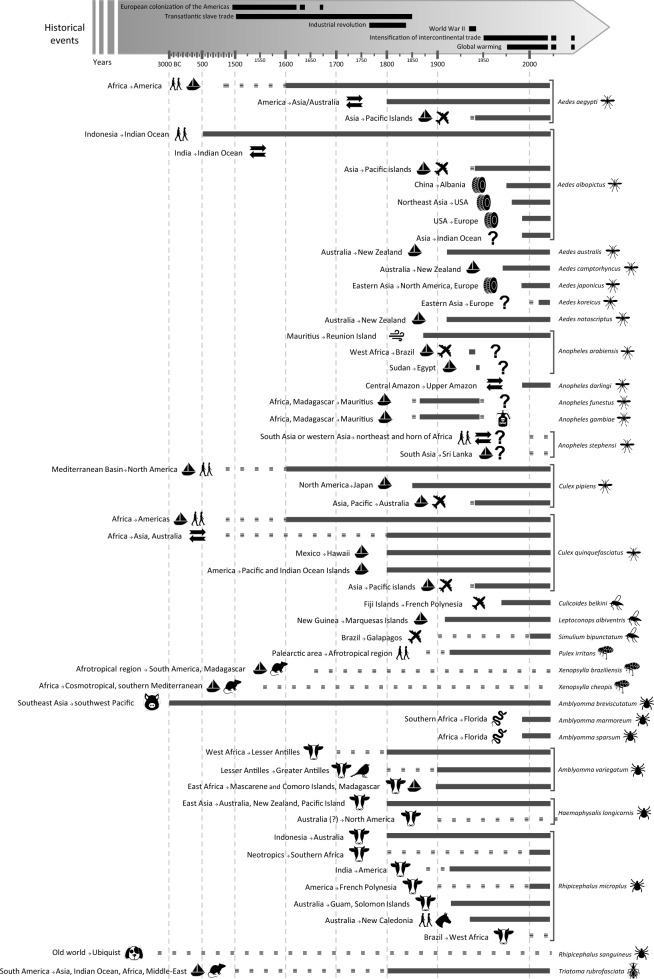

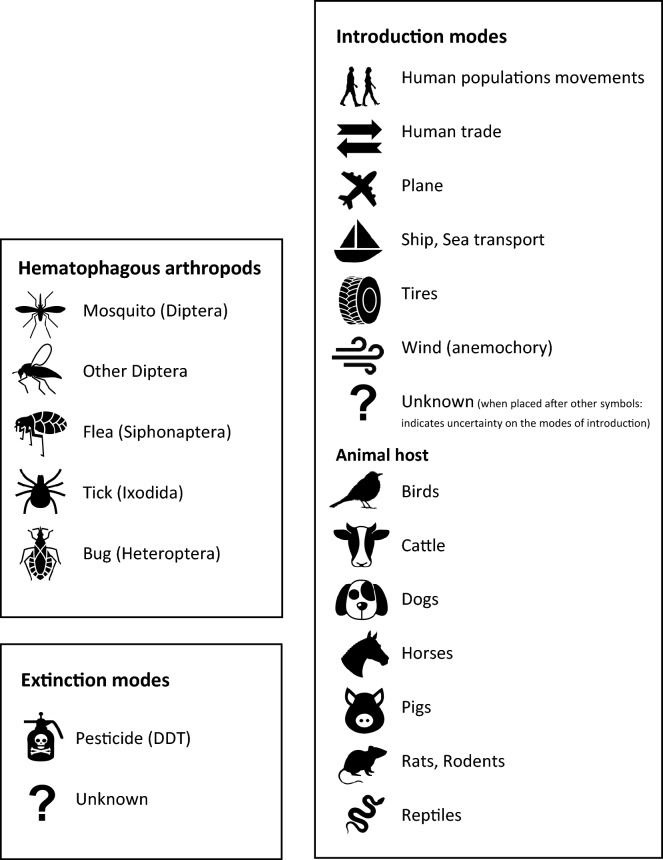
Synthetic representation of the historical trajectory of the different waves of dispersal of hematophagous arthropods. The gray arrow at the top of the figure represents the years from 3000 BC to present time. The solid black lines within the arrow relate to important historical events linked to the dispersion of species (the dotted parts show events either ongoing or with no clear end). Hematophagous arthropod species are identified on the right side of the figure, followed by a symbol representing the taxon (see symbols legend). Each line represents a different wave of dispersal (species with several waves are shown with brackets). For each wave, the geographical details are written following the format “Native Area Alien Area.” Symbols represent the mode of introduction (mechanical and/or via animal hosts) for each wave (see symbols legend). The solid black line represents the time frame of the species’ presence in the alien area. The dotted parts at the end of the lines show either uncertainty in the establishment/extinction dates (particularly when the entire line is dotted) or introduction/extinction occurring during an extended period of time. When the species has become extinct in the alien area, a symbol at the right end of the line represents the mode of extinction (see symbols legend)

By definition, organisms are considered to be alien when they occur in a novel region as a result of human transportation and activities [40]. For hematophagous arthropods, the majority of introductions have occurred by seas [41]. The transport and arrival of alien organisms by humans greatly increased during the fifteenth and sixteenth centuries through intensification in commercial trade, agricultural practices, decrease of forest cover and of fallows [42]. This land use change and intensification of trade and transport of goods and materials by sea, including the transport of livestock, also impacted hematophagous arthropods, as many are closely linked to humans or livestock for completing their life cycle. Changes in the geographic distributions of several disease-vectoring arthropods were recorded during that period, including the mosquitoes *Aedes aegypti*, *Ae. albopictus*, *Culex pipiens* and *Cx. quinquefasciatus*, the ticks *Amblyomma breviscutatum* and *Rhipicephalus sanguineus*, and the kissing bug *Triatoma rubrofasciata* (Fig. 1, Additional file 1: Appendix S1). Among these species, the invasion pathways of *Ae. aegypti* (see Powell et al. [43] for a comprehensive review of the invasion history of this mosquito species) and members of the *Cx. pipiens* complex (i.e. *Cx. pipiens*, *Cx. quinquefasciatus*) have been intensively studied and monitored.

Both *Ae. aegypti* and the *Cx. pipiens* complex are associated with key historical events related to international trade and colonialism, such as the European colonization of the Americas and the slave trade. Accordingly, both of these hematophagous arthropods and their associated viruses likely arrived by ship during that period, though often the pathogen arrived after the mosquito [31] (Fig. 1). Historical outbreaks of yellow fever (vectored by *Ae. aegypti*, a species native to Africa) in the New World were recorded as far back as 1648 and as far north as New York City [44, 45]. Epidemics of West Nile virus from *Cx. pipiens* started later than yellow fever (from 1999 in North America [46]).

## Long distance transport and livestock trade facilitated tick translocations

Unlike mosquitoes which feed only during the adult stage, ticks require blood meals to progress across each of their life history stages following hatching, and their full life cycle takes comparatively longer (up to 3 years), while constrained near the ground in the terrestrial realm. Consequently, the means of introduction of alien tick species differ from those of mosquitoes, which have a more complex aquatic life history stage. For ticks, introduction is often linked to specific vertebrate hosts used in agriculture. The translocation of several tick species (*Amblyomma variegatum*, *Haemaphysalis longicornis* and *Rhipicephalus microplus*) dates back to the eighteenth or nineteenth century. For *A. variegatum*, introduction to the Caribbean region was due to the human-mediated transport of their cattle hosts from west Africa [47], and introduction of other tick species was associated with the global trade of livestock [48].

### Booming invasions with the early nineteenth century globalization

The next significant wave of disease vector species translocations occurred with the increase in air transport and traffic eventually connecting almost all continents by the early 1900s (Fig. 1). For example, in 1930, *Anopheles arabiensis,* belonging to the *An. gambiae complex,* was first observed in Brazil where it quickly established and expanded in the decades that followed its introduction [49, 50]. The vectorial capacity of this mosquito for *Plasmodium falciparum*, combined with rapid population proliferation, caused significant epidemic outbreaks resulting in the death of 14,000 people in Brazil during 1938–1939 [49]. It was nonetheless successfully eliminated from the continent through an integrated program, but one which relied overwhelmingly upon larval control [51]. The rise in air travel in the 1900s was also associated with range expansions of known and already established alien species, in addition to novel introductions [52, 53] (Fig. 1). For instance, *Ae. aegypti* was first recorded in Asia and Australia during the nineteenth century, despite being known as an invasive alien species in America since the fifteenth-seventeenth centuries [41]. Most of the other invasive mosquito species, *Ae. albopictus* [54], *Cx. pipiens* [55] and *Cx. quinquefasciatus* [56], were also and are still propagated by air transport nowadays. Similarly, *Culicoides belkini* was introduced in French Polynesia and further expanded its range within a few decades in the islands of the archipelago [57]. As a consequence, the expansion and re-emergence of associated vector-borne diseases has also increased [58].

### The Anthropocene Era: human-made artificial habitats as significant sources of hematophagous arthropods and their associated diseases

Most alien mosquitoes are highly adapted to synanthropic contexts and are highly efficient in exploiting human-made water containers for larval breeding sites. In general, life history traits often assist the invasive potential of hematophagous arthropods. For instance, *Ae. albopictus* is able to produce drought- and freeze-resistant eggs. Human-made microhabitats have amplified translocation rates and establishment possibilities for mosquito species with such adaptations. The worldwide development in the trade of used tires since the 1950s, and of certain ornamental plants, such as lucky bamboo (*Dracaena sanderiana*) [2], have become the principal points of entry within Europe and other continents. This is particularly true for temperate species capable of producing dormant eggs in discarded automobile tires, like for *Aedes* spp. [59, 60]. For example, used tire exports from countries such as Japan have been responsible for the introduction of *Ae. albopictus* to countries such as the USA [61] and have been regarded as an important introduction pathway for this species, alongside sea transport, plant translocations and ground vehicles [62]. For ornamental plants, lucky bamboo was identified as an important introduction pathway in Belgium, and plant nurseries were subsequently targeted for control measures [63]. Other goods have been implicated in the transportation of *Ae. albopictus*, such as repatriated military equipment from Vietnam and stone fountains from China [64].

### Prediction and prevention of future invasions

Connectivity among cities, international trade and anthropogenic environmental disturbances are likely to increase in the future, providing novel opportunities and generating ecological conditions propitious for the expansion or new introduction of hematophagous arthropods that may vector disease (Fig. 1, Additional file 1: Appendix S1), which can subsequently proliferate in their new ranges [65]. The “Belt and Road Initiative” (BRI) proposed by China is a prominent example, as this unprecedented global development project may promote further invasions by hematophagous arthropods. In particular, 14 hotspots in the world have been identified as being at considerable risk of invasions [5], and these mainly fall along the economic corridors proposed in China’s BRI project, thus placing 68 countries at high risk of invasion [5]. While Liu et al. [5] focused on the potential risk of invasion by terrestrial vertebrates—and did not consider invertebrates such as hematophagous arthropods—we suggest that hematophagous arthropods will also increase their range in response to such an increase in connectivity. This assumption is supported by the example of *Ae. albopictus*, whose dispersal is amplified by road traffic [66, 67].

After arrival, expansion from introduction points can be further enabled by passive transportation of mated females through road traffic [67], with petrol stations and highway parking lots, as well as seaports, railway stations and airports, subject to heightened surveillance [64]. Recent mathematical models have found that better prevention (e.g. surveillance and early detection) could have made multi-billion dollar savings for *Aedes* spp. alone, by mitigating future damages and control efforts [68]. However, this particular focus on mosquitoes involves only a subset of potential proactive approaches to manage invasions, which remain to be optimized for a range of taxonomic groups (and also see Box 1).

Box 1—Novel techniques for the surveillance of alien insect species In attempts to reduce the risks and impacts of vector-borne diseases, increased surveillance relying on up-to-date methods and using cutting edge technology is required (also see [69] for more information on the joint plan of action and the accrued needs for surveillance). Xenomonitoring [70], i.e. a disease surveillance technique relying on molecular genetics to detect the DNA or RNA of a pathogen or parasite of human or animal health importance in hematophagous arthropods, should be more systematically encouraged to detect early stages of arthropod invasion. Citizen-science approaches, using the perception of mosquito nuisance reported by citizens as a potential indicator for malaria, dengue and other disease vector hotspots [71], should also be developed to produce global health risk indices at low costs. Citizen science findings can also complement official and systematic surveillance to improve detection of alien species [72]. Recent data collection applications (e.g. Mosquito Alert, http://www.mosquitoalert.com; GLOBE Observer, https://observer.globe.gov/; Invasive Mosquito Project, http://www.citizenscience.us/imp/) involving cutting-edge technology have been implemented to monitor mosquitoes and models have been developed to partly automate invader identifications [73]. Recently, the near-infrared spectroscopy (NIRS) technique has been successfully used to detect Zika and chikungunya infection in dead *Ae. aegypti* female mosquitoes [74], providing a rapid and cost-effective arbovirus surveillance tool with high accuracy levels (> 90%). Moreover, a better understanding of invasions and emerging diseases associated with hematophagous arthropods provides a study system that will inform on the management of other pandemics, such as COVID-19, given the obvious links between invasion science and infectious disease transmission [75].

For hematophagous arthropods in general, the development of biosecurity toolkits relying on semiochemical methods should be developed, so that innovative multi-species traps with combinations of attractants can be designed [76, 77]. By additionally considering the use of (multi-)lure blends, as experienced for cerambycid species [78] or bark beetles [79], for instance, we suggest that this method is a promising avenue for the cost and time-effective surveillance of future invasions.

## Climate change in shaping invasions, diseases and socioeconomic impacts

### Climate change and the opening of novel thermal niches

Climate warming may have a major role in fostering hematophagous arthropod invasions by increasing the number of favorable thermal niches for their development, including towards formerly cold and restrictive environments, such as at higher latitudes and altitudes [80]. Even if the outcomes of future distribution forecasts should be considered carefully and include different scenarios, Carvalho et al. [81] showed that many disease vectors are expected to expand their range, especially towards the poles. As a consequence, climate change is likely to worsen health risks by increasing zoonotic diseases, transmission and disease vector population growth after the arrival of alien hematophagous arthropods (e.g. *Ae. albopictus* and *Ixodes ricinus* [82]) (also see Box 2 for additional information).

For the highly invasive yellow fever mosquito *Ae. aegypti*, Iwamura et al. [9] suggested an increasing global suitability for life cycle completion of + 1.5% (in terms of periods suitable for mosquito development) per decade between 1950 and 2000, with that trend predicted to accelerate and increase by + 3.2% per decade, and even up to + 4.4% per decade, by 2050. In southern Europe, future climatic predictions suggest a northward expansion of *Ae. aegypti* from the limited remnant population in the Black Sea area, introduced over a century ago [83], and particularly to container ports of the Alboran, Balearic and Aegean Sea areas [84]. For *Ae. albopictus* and the Asian rock pool mosquito *Aedes japonicus*, Cunze et al. [85] predicted differential habitat suitability and range shifts under future climate change scenarios up to 2080 in Europe, with *Ae. albopictus* expected to expand its range and *Ae. japonicus* expected to contract its range. *Aedes albopictus*, native to the tropical and temperate forests of southeast Asia [86], has already extended its distribution range to all temperate zones of the planet in response to global warming and the increase in human-mediated trade and transport. The proliferation of *Ae. albopictus* in temperate regions is partly due to its eggs being able to enter diapause during winter. Eggs do not hatch until the following spring, when they likely exist in a quiescent state until environmental conditions become favorable for hatching [87]. Given that climate is expected to warm at least by 3 °C by 2100, cold-related stresses will disappear in many habitats of the world, and *Ae. albopictus* will no longer have to go through the winter diapause stage to survive [88].

Similarly, we suggest that the lessening of thermal barriers could permit *Ae. aegypti* and tick species, such as those from the *Ixodes* genus, to expand their range into warming temperate regions, increasing disease risks [89]. Importantly, such predictions can present substantial heterogeneity, and species-specific differences may occur, as reported for tick species dynamics [90], and for tick-borne and mosquito-borne diseases [91]. While mosquitoes can be affected by extreme weather events and climatic variability in the short term, ticks will respond to climate change through long-term changes in their spatiotemporal occurrence [91].

Many alien hematophagous arthropods, currently introduced but apparently not spreading or causing impact yet [92, 93], will likely transition to invasive species in the future, owing to the concept of invasion debts (i.e. time lags to alien species spread and impact following arrival [94]). Indeed, the impact on the environment of an alien species recently introduced to a new ecosystem, though initially benign, can be the premise of a proliferation detrimental to the invaded habitats or society. In this context, the assessment of population dynamics and distributions of hematophagous arthropods is key, as this then allows development of reliable predictions for the effects of climate change on disease transmission, and thus robust estimates of the populations that are at highest epidemiological risks. Modeling efforts should therefore concentrate on the worldwide projections of vector-borne diseases under different future scenarios involving climate change, pollution and urbanization. This would mean that regions, including polar and high altitude areas, and populations at higher epidemiological risks can be better equipped, whilst currently there is low awareness, unprepared medical systems and immune naiveté of the population to the disease [95]. Furthermore, the increasing rates of sea level rise caused by global warming will significantly increase the area of coastal marshes by incursions inland [96]; those coastal marshes are historical habitats for mosquitoes [97] and their expansion would thus foster their re-emergence alongside diseases.

Finally, the risk of native vertebrate species extinction due to alien exotic pathogens, including alien hematophagous arthropods, is particularly acute within insular systems harboring endemic fauna composed of immunologically naïve species. For example, the mosquito *Cx. quinquefasciatus*, a vector of avian malaria (*Plasmodium* sp.), represents a serious threat to the Galapagos and Hawaii bird diversity [34, 35].

Box 2—The concern for climate change heightened vector-borne diseases Range shifts of native species tracking climate change [10] rely on different processes and mechanisms as compared with long-distance transportation of species by humans [24, 98–100]. Lindgren et al. [101] reported a northward expansion of the range limit of *I. ricinus*, a disease-transmitting tick, between the early 1980s and mid-1990s in Sweden, which they related to milder winters and extended spring and autumn seasons. Lindgren and Gustafson [102] further demonstrated that warming has increased the incidence of tick-borne encephalitis in central and northern Sweden during the same period. More recently, a multi-source analysis has reported both latitudinal and elevational range shifts of *I. ricinus* at its northern distribution limit in Norway [103]. In Sweden, most of the range expansion occurred north of 60°N, where the tick's coverage area doubled from 12.5% in the early 1990s to 26.8% in 2008, reaching 66°N due to a milder climate combined with the spread of roe deer (*Capreolus capreolus*) [104]. All these reports suggest a growing incidence of tick-borne encephalitis and other tick-borne zoonoses (e.g. Lyme disease) in the future, as far as 70°N, as future climate will become even more favorable to *I. ricinus* in northern Scandinavia [105]. Similarly, in northeastern Canada, Ogden et al. [106] have projected that future climate change will result in a northward shift in the range of the Lyme disease vector *Ixodes scapularis*. Consequently, there has been a call for public health responses to threats of emerging infectious diseases in the Arctic [107]. For instance, Parkinson and Evengård [107] recommend enhancing the public health capacity to monitor Lyme disease and tick-borne encephalitis in areas across the Arctic, at the margins of regions or countries known to support animal hosts, reservoirs and insect vectors of disease, and where climate change may promote their geographic expansion.

In the tropics, malaria—the most prevalent mosquito-borne disease—is becoming more prominent in the highlands of Ethiopia and Colombia because of the upward range shift of *Anopheles* mosquitoes along elevational gradients of mountainous ecosystems in recent decades [108]. Hence, hematophagous arthropod species, be they currently native or alien, can go beyond their native range limits and have, under anthropogenic climate change, harmful impacts on human health. These threats are not only more pressing in the Arctic, where climate is warming at four times the global rate, but also in more temperate regions through poleward migrations from the tropics. The invasion of urban areas worldwide by synanthropic disease vector mosquitoes further increases the risk for rapidly transforming local epidemics into global pandemics [109], once pathogens are introduced in immunologically naive and strongly interconnected urban populations, as observed during the Zika crisis.

As another example, the anopheline species *Anopheles labranchiae* can be regarded as particularly threatening to human populations. Originating from northern Africa, *An. labranchiae*, a member of the *Maculipennis* subgroup, established in many countries bordering the Mediterranean Sea, with the serious risk of reintroduction of the most severe form of malaria [110–112]. The larvae of this mosquito develop in the stagnant waters of irrigation canals, ditches, marshes and rice fields [113]. The adult females are extremely prolific feeders upon humans, especially at night [114]. Individuals overwinter in animal sheds as well as in natural sites, such as rocky crevices or tree holes [113]. The larval bioecology of this mosquito, the anthropophilic behavior of the females and their ability to enter diapause during the winter, make *An. labranchiae* a malaria vector of great adaptability to various environments. All over southern Europe and northern Africa, a number of resident anopheline mosquito species could possibly act as disease vectors, but *An. labranchiae* is the leading candidate by virtue of its historic role in the transmission of *Plasmodium falciparum*, *P. vivax* and *P. malariae* [112]. Because of this high risk and the ubiquitous nature of this species, we therefore suggest thorough and continuous distribution monitoring to detect potential spread, especially in southern Europe and beyond its range limit in northern Africa, where it has not been identified yet, and where the risk of spreading malaria is high.

### Climate change will worsen economic costs

The impact of hematophagous arthropods on human societies is often assessed either as an economic cost (in monetary currency) or in disability-adjusted life year (DALY), which represents the life expectancy lost because of the burden of insect-borne diseases. While the costs in terms of human lives and suffering should be sufficient to warrant effective measures against the spread of hematophagous insects and the pathogens they carry [115], it is in fact the huge economic costs incurred by the spread of hematophagous insects that ironically provide powerful and more tangible metrics for actions by international authorities. Economic costs therefore appear to be more straightforward to use for synthetic and applied purposes, and especially in the context of invasive alien species [20], including hematophagous arthropods [4, 23].

Economic studies on invasive hematophagous arthropods revealed heterogeneous, either direct (e.g. [116]) or indirect (e.g. [117]), cost figures worldwide. However, global syntheses are still scarce and evidenced a spatially biased repartition of these costs (e.g. [116]). Similarly, the number of reported vector-borne diseases considered in such existing studies is very limited and underrepresents the reality, with dengue representing up to 84% of total health costs, the West Nile virus representing 15% and Chikungunya and Zika together representing < 1% [18]. The existing knowledge reveals that only 15% of the economic costs incurred by hematophagous arthropods are devoted to their control (6% being of unknown use), the remaining being medical care (52%) or both medical care and control (27%) [18, 20]. The part of the control that is allocated to biosecurity and early detection of newly introduced species remains unknown (information retrieved from the public database InvaCost v5, https://doi.org/10.6084/m9.figshare.12668570.v5). This is surprising, as a recent economic evaluation of the cost-effectiveness of disease vector control in six countries showed that control expenditures would cost less than the outbreak response, with populations reduced by up to 90% [118]. Warmer conditions will likely increase mosquito populations and abundance, in turn increasing biting rates. As incubation periods for the parasites and viruses they carry are also temperature-dependent, climate change will certainly contribute to a switch from occasional to frequent disease outbreaks (for instance, malaria and dengue), in parallel to providing more regions where the vectors and their associated diseases could arrive and spread. Almost 2 decades ago, Cumming and Van Vuuren [119] were already pointing out the significant impact that climate change could have on the occurrence of tick-borne diseases and the extremely high economic importance this could have.

Rapid response to eradicate invaders post-invasion as well as the implementation of effective biosecurity control pre-invasion (i.e. biosurveillance and intensive monitoring) are up to ten times cheaper for mosquito-borne diseases than waiting and paying for accrued damages [120]. A recent modeling study using logistic response curves to resemble impact dynamics showed that for the *Aedes* genus, current management delays of 55 years have led to an additional cost of US$ 4.57 billion that could have been avoided, showing the crucial importance of acting early against future invasions and other global changes [68, 121]. With climate change projections over the next 3 decades likely to increase the area occupied by current arthropod invaders by 18%, the need for better biosecurity control to mitigate hematophagous arthropod nuisance biting and disease has never been stronger [7]. Moreover, knowledge needs around the potential synergistic links between climate change and invasive alien species impacts are pervasive, both concerning ecosystems and economies [25]. Yet, even if additional investigations on the influence of temperature on disease transmission are required, there are lines of evidence suggesting that vectorial capacity and vector competence could be increased in certain circumstances [122], further increasing medical costs.

Even aside from direct health costs, invasions by hematophagous arthropods can cause substantial impacts through nuisance biting that affects recreational and real estate values which yield an economic return [123]. As lagging effects in population dynamics are the rule during the invasion process, invasive populations may grow undetected for many generations before reaching a threshold where they become abundant enough for economic damages to occur or be noticed. Indeed, Cuthbert et al. [124] found that invasion costs relate positively to the time duration a species has been present, signaling that failing to respond rapidly could prove more costly in the future.

The global economic burden associated with invasive hematophagous arthropods that vector diseases is most likely underestimated, often excluding indirect economic impacts on productivity and income [125], tourism [126], blood-supply system [127], personal protection [128] and quality of life [129], not to mention the contribution of each disease to DALY, which seldom include monetary values [18]. With climate change likely to provide opportunity for new invasions and exacerbate current ones, future economic impacts from hematophagous arthropods that vector diseases will likely rise substantially.

## Hematophagous arthropod invasions in the anthropocene

### Urbanization and heat islands

Four and a half billion people currently live in cities, about 55% of the world population [130]. This represents a growing opportunity for the development of invasive mosquito populations [131, 132] due to: (i) human-generated dumping sites in both public and private spaces; (ii) the deterioration of roads; (iii) the pollution of surface and ground waters, which some arthropod disease vectors (e.g. *Cx. pipiens*) use as resources. Growing urbanization also results in large numbers of construction sites, often offering additional microhabitats for the proliferation of mosquito populations [133, 134]. Mosquito communities in urban areas have also been shown to be less diverse, but also more abundant, being dominated by a few species that are adapted to develop effectively in artificial environments [135]. In cities, larvae of most alien mosquitoes, including *Ae. aegypti*, *Ae. albopictus* and *Cx. pipiens*, largely benefit from the presence of scrap tires, plates under flower pots, clogged roof gutters, cement tanks, metal pots, cemetery urns and many other water storage containers [136, 137]. In these human-made habitats, often associated with private gardens and urbanized areas, water collections support larval development and are available in similar forms worldwide, thus representing a significant predictor of the presence of *Ae. albopictus* [138]. Moreover, they often contain a smaller suite of natural enemies due to their frequent inability to colonize very small, often transient, urban aquatic habitats.

In urban environments, females of *Cx. pipiens* (*Cx. pipiens* and *Cx. quinquefasciatus*) often oviposit in the polluted waters collected in sections of ditches and many other sites contaminated by urban effluents [139, 140]. There is growing evidence that major human malaria vectors in Africa, and especially *An. coluzzi* and *An. arabiensis* within the *An. gambiae* complex, are also thriving in rapidly expanding urban metropoles, where non-specific insecticide resistance mechanisms selected in agricultural settings may promote adaptation to polluted waters [141, 142]. *Aedes aegypti* and *Ae. albopictus*, two other urban mosquito vectors of dengue, chikungunya and Zika viruses, more frequently oviposit in clear, domestic and peridomestic water collections before their inundation from rainfall [143]. Of note, the recent invasion of major cities in easternmost Africa (i.e. Djibouti, Ethiopia and Sudan) by the Asian urban malaria vector *Anopheles stephensi* breeding in water tanks has already given rise to severe malaria epidemics and is potentially threatening over 126 million people in its novel predicted range in Africa [144]. The presence of larval microhabitats for *Ae. aegypti*, in the form of unsealed urban water storage containers, can also greatly improve connectivity among mosquito populations and thus favor the spread within urban environments [145]. Moreover, mathematical simulations have evidenced that high unsealed tank densities and the presence of non-compliant tanks can bolster the invasiveness of the species by reducing habitat fragmentation [146].

While not necessarily causing a difference among native and alien mosquito populations, urbanization may have different effects on the distribution of mosquito species. For example, in Guangzhou (China), urbanization providing human-made container habitats is beneficial to *Ae. albopictus* [147] in the absence of domestic populations from *Ae. aegypti*. Conversely, the more heat-sensitive species *Ae. japonicus* suffers from urbanization in Fukuoka city (Japan) and especially from the urban heat island effect [148]. Furthermore, for *Aedes koreicus*, human population density has been found to negatively affect mosquito abundances, suggesting that they rely on other blood meal sources [149]. However, the urban heat island effect in cities can also be beneficial, by causing shifts in phenology, promoting more rapid development associated with increased temperature and causing earlier seasonal population peaks in temperate areas [135] as well as acting as stepping stones to foster alien range expansion. Notably, the population density of the alien species *Ae. albopictus* in its invaded range can be inversely related to the distance to the nearest vegetation border [150], almost suggesting a niche inversion (i.e. reversal of niche characteristics between native and invaded regions) compared with the original habitat of the same species within its native range, as invasive alien populations can prefer urban areas over vegetated ones. Human environments can also foster the potential evolution of sub-populations, exemplified by the supposed “London Underground” subspecies of *Cx. pipiens*, with surface and subterranean populations genetically distinct and displaying different reproductive and feeding behaviors [151] (but see [152]).

### Environmental pollution

The effects of light pollution, i.e. artificial light at night in urban areas, on the life cycle and physiology of mosquitoes and other hematophagous arthropods that vector diseases are also becoming a growing research topic, possibly further strengthening the invasion potential of alien species. A recent study indeed evidenced the alteration of the seasonal phenology of *Cx. pipiens*, whose females have prolonged reproduction and biting seasons when exposed to urban light pollution [153], thus increasing the proliferation of alien mosquitoes and their epidemiological significance. It is thus expected that the ongoing worldwide urbanization, and the increasing problem of water storage by households in areas exposed to light pollution at night, will further support mosquito proliferation.

In agricultural lands, the fertilization of rice and vegetable crops usually takes place in warm and sun-lit waters, already propitious to the development of *Anopheles* and *Culex* mosquitoes. Importantly, NPK fertilizers (N for nitrogen, P for phosphorus and K for potassium) strongly attract gravid females of mosquitoes searching for nutrient-rich oviposition sites [154–157]. While the NPK fertilizer is not directly assimilated by the mosquito larvae, the three minerals enhance the development of bacteria, algae and fungi, increasing the food biomass of the breeding sites [158]. The larvae of mosquitoes exploit this additional biomass to proliferate. Laboratory studies found the survival rates of *Ae. aegypti* and *An. gambiae* in waters contaminated by fertilizers to be two-to-three times as great as in the uncontaminated waters [159, 160]. Direct nutrient inputs from grazing cattle also increase mosquito proliferation [161].

Finally, in regions of intensive monoculture, where pests and parasites represent a real threat to plants, there has been a sustained use of fungicides, herbicides and insecticides. Those substances, used repeatedly, favor the emergence of multiple lines of resistance among pests and mosquitoes, while causing the decline of predatory insects, amphibians, reptiles and fishes [162]. In sum, the growing human population requiring increased agricultural productivity will lead to increased insecticide resistance and proliferation of mosquito populations associated with these activities [140].

## Social ethnology to improve health and environment quality

### Transformational change and paradigm shift in perceptions

Until recently, the sociological and ethnological literature on hematophagous arthropods tended to fit into two distinct sectors: ‘health specialists’ dealing with disease and epidemic risks to humans and ‘environment specialists’ dealing with the relations between insects and their ecosystems [163, 164]. This ‘health’ versus ‘environment’ distinction can be paralleled with a ‘tropical’ versus ‘temperate’ contrast in the geographical space. In tropical regions, populations are still heavily burdened by the diseases that hematophagous arthropods transmit, with mosquitoes remaining one of the deadliest disease vectors. That is despite the considerable progress achieved in recent decades, owing to the distribution of insecticide-treated mosquito nets for malaria control (http://www.who.int/malaria/en), in particular. Until the 1990/2000s, in temperate regions, the health issues related to hematophagous arthropods were considered a thing of the past. In this context, research rather focused on environmental issues and on perceptions towards these insects, the identity attachments they may be involved in and the socio-technical controversies related to their management—particularly those concerning comfort-based mosquito control policies [163, 165–167].

In recent decades, however, several factors have called into question this tropical/temperate division. In temperate regions, the introduction of alien vector species, which subsequently became invasive, exposed populations to new epidemic risks and high levels of nuisance. The case of *Ae. albopictus* is particularly representative of the crossover between environmental and health issues. The introduction of this mosquito species into southern Europe in the early 2000s disrupted the vernacular taxonomies associating mosquitoes with polluted urban places (i.e. sewage) or to wilderness (i.e. wetlands). *Aedes albopictus*, conversely, prefers habitats in proximity to domestic spaces in clear waters and consequently has provoked reactions of denial or responsibility shifts among the human population [168]. In addition, the ability of *Ae. albopictus* to proliferate and harm human health challenges ecological belief in European populations, further widening the gap between their ecological discourses and practices [169, 170]. This ‘ladybird syndrome’ [171] leads the same individuals to declare themselves highly sensitive to the protection of nature, while demanding the eradication of *Ae. albopictus* by a biocide. In a further paradox, human populations use the health risk argument to justify their requests for mosquito control, while expressing relatively little concern about the occurrence of an epidemic of dengue, chikungunya or Zika in their region [172].

As populations gain awareness of the adverse ecological and health effects of biocides, the increasingly ineffective insecticide treatments, which apply to mosquitoes that vector disease first and foremost [173, 174], are the subject of burgeoning protests. These environmental concerns are accompanied by growing aspirations for genuine consultation with local populations, for instance, prior to the testing of genetically modified mosquitoes in various southern countries [175]. In this nexus between environment and health, the progressive changes in the wording defining Lyme disease transmitted by ticks of the *Ixodes* genus—from infectious, to vectorial and then zoonotic—have exemplified the progressive ecologization of health issues [176]. In other words, human populations are increasingly aiming to improve environmental safety by limiting human-made environmental threats, including the reconsidering of management of invasive hematophagous vectors of disease with chemicals. Requests for integrating environmental protection measures in the discussions around management of outbreaks of invasive hematophagous arthropods have gained particular traction in tropical regions, where ecological framing of health issues has been progressively paired with democratic demands. In recent decades, the constantly increasing connection between environment and planetary health has contributed to the development of the now well-known One Health approach [177], whose definition was revised in 2022 [178].

### Co-constructive and deliberative approaches

The recent sociological and ethnological literature on hematophagous arthropods is increasingly considering the interactions among human, animal and environmental health [172, 179–182]. Novel research questions will arise from the comparative analyses of the emergence and re-emergence of ecological and/or health concerns as well as shifts in priorities in diverse socio-ecological contexts. These will pave the way for novel generations of sociologists and ethnologists to bridge together ecologists, entomologists, virologists and other public health and environment specialists to securely anchor their working hypotheses on solid biological roots. In turn, biologists interested in hematophagous arthropod invasion biology and its ecological and evolutionary tenets should benefit from strengthened interactions with social scientists. This will allow them to better integrate sociological and ethnological dimensions when performing risk assessment analyses and/or formulating scenarios and hypotheses for vector evolution as well as associated disease emergence and spread [183]. Altogether, this will increase uptake of research results by non-academic stakeholders, including civil societies, regulators and authorities, by providing data and recommendations for evidence-based decisions. The importance of considering biodiversity and environmental value pluralism in management action discussions has been recently advocated by Meinard et al. [184], who proposed solutions aimed at implementing consensual plans for biological invasion mitigation efforts.

## Conclusions

1. The worldwide invasion of hematophagous arthropods is a longstanding and pervasive problem, which is predicted to further increase with climate change. In addition to global climate change, our review highlights the importance of shipping and air traffic, the transport of vertebrates (i.e. livestock and pets) and worn tires as prominent factors and sources assisting the introduction of hematophagous arthropods that vector diseases. The management of invasive hematophagous arthropods should be more proactive and consider expected changes in socioeconomic activity patterns worldwide so that new potential pathways and species source pools for introduction could be identified. Given the focus on a few groups here (principally mosquitoes and ticks), future works should consider invasion dynamics and impacts of additional, understudied hematophagous arthropod taxa.
2. Several hematophagous arthropods have exhibited strong ecological niche shifts, including niche inversion phenomenon, during the invasion process. The opportunistic development of populations in a large variety of human-made microhabitats is increasingly reported. Here, we alert decision-makers and politicians to the knowledge gaps and growing risks posed by accelerated worldwide urbanization and interconnectivity, which could further support mosquito proliferation and adaptations to human-modified environments.
3. Surveillance systems and strong political commitment are required to set-up and maintain proactive disease prevention programs and preparedness for rapid responses to outbreaks.
4. Finally, social-ethnological approaches represent a valuable perspective for setting mitigation measures aiming at improving health and environments in the context of proliferations of hematophagous arthropods that vector diseases. By encouraging the dialogue between experts of health and environmental topics, including epidemiologists, policymakers and researchers, and considering social perception, the chances of exploring issues related to encounters between hematophagous arthropods and humans, including new encounters, would improve. Beside the significant health and economic concerns posed to human populations, our review demonstrates several nuisances to wildlife, highlighting the importance of placing the management of these arthropods in a wider biodiversity context, i.e. by the consideration of a multispecies well‐being.

### Supplementary Information

**Additional file 1:** List of invasive hematophagous arthropods (species name). For each species, the following information, whenever available, is mentioned: native area (i.e. the known native geographic zone of the species), the invaded area, date or period of introduction, the date of extinction, the introduction pathway, the diseases / pathogens that are hosted by the species, the ecological impact, the description of the larval and adult habitats, and for ticks and fleas the mammals vectoring the species.

## Data Availability

No data were collected for this study (review). All data and information synthesized in the review are already published and publicly available, and those publications are properly cited in this submission.
